# Examination of Daytime Sleepiness and Cognitive Performance Testing in Patients with Primary Insomnia

**DOI:** 10.1371/journal.pone.0100965

**Published:** 2014-06-24

**Authors:** Hong Liu, Dexi Wang, Yun Li, Zhe Li, Ying Zhang, Fei Lei, Lina Du, Xiangdong Tang

**Affiliations:** 1 Sleep Medicine Center, Mental Health Center, Translational Neuroscience Center, West China Hospital, Sichuan University, Chengdu, Sichuan Province, China; 2 Department of Internal Medicine, First People's Hospital of Yibin, Yibin, Sichuan Province, China; 3 Department of Neurosurgery, First People's Hospital of Yibin, Yibin, Sichuan Province, China; 4 Department of Psychology, Dalhousie University, Halifax, Nova Scotia, Canada; Peking University, China

## Abstract

**Objective:**

While individuals with insomnia consistently complain of cognitive impairment, previous studies on the effect of insomnia on objective measures of cognitive function have obtained ambiguous results. The relationship between daytime sleepiness and cognitive manifestations in insomnia patients is not clear.

**Methods:**

Thirty-six primary insomnia patients (PIPs) and 26 good sleep controls (GSCs) with age and gender matched manner were included in the study. Participants underwent an overnight polysomnography followed by a multiple sleep latency test (MSLT) and an examination of the attention network test (ANT). ANT reflected three attentional networks including alerting, orienting and executive control. According to whether accompanied with excessive daytime sleepiness (EDS), the insomnia group were subdivided into PIPs with EDS (n = 12, score on MSLT<10 min) and PIPs without EDS (n = 24, score on MSLT≥10 min).

**Results:**

PIPs only performed worse on executive control function than GSCs in ANT. PIPs with EDS had longer overall reaction time (RT) related to PIPs without EDS. Further analyses with Pearson correlation analysis showed a significant negative correlation between the overall RT and MSLT latency in insomniacs (*r* = −0.444, *p*<0.01), whereas no such correlation was found in controls.

**Conclusions:**

Results suggest that PIPs do show executive control function deficits compared with GSCs. Daytime sleepiness in terms of MSLT latency was associated with poor cognitive manifestations in patients with insomnia.

## Introduction

Insomnia is a widespread health issue throughout the world, referring to the complaints of difficulty in nighttime sleep and associated daytime functioning problems, such as fatigue, sleepiness, mood disturbances, and cognitive impairment [1]. For patients with insomnia, the reductions in the capacity of concentration and memory are among the most often seen symptoms, but the laboratorial examinations did not provide unequivocal evidences of global cognitive dysfunction [2], [3]. A Meta-analysis suggested that individuals with insomnia performed more poorly on tasks assessing working memory, episodic memory and selective attention, but not in the other components of attention (e.g., alertness, divided attention) [4]. Compared with controls, only 20%–25% of studies reported that insomnia suffers have cognitive impairment, based on objective neuropsychological performance [5], [6]. The negative findings in the objective evaluation for cognitive impairment among insomnia may be related to the heterogeneity of subjects and use of insensitive measures to detect mild impairment in insomniacs.

The various tests of cognitive function are widely categorized into memory, working memory and attention. An efficient attention is essential for other cognitive components [7]. Neuroimaging studies have demonstrated that insomnia suffers exhibited decline in metabolism in regions associated with cognition and the neuronal basis of attentional processing appears to play the central role in terms of large-scale neuronal networks [8]–[10]. Based on the hypothesis of anatomical network model of the human attention system, Posner and Petersen [11] proposed that attention can be broken down into three networks of alerting, orienting, and executive control. Alerting is the ability to prepare and sustain a vigilant state; orienting refers to shifting attention in order to select information; executive control is defined as resolving conflict among responses. The Attention Network Test (ANT) was developed to reflect those three dimensions of attentional functions within a single task [12]. Since the ANT was established, it has been used to uncover attention deficits and various clinical disorders, including mild cognitive impairment [13], attention deficit hyperactivity disorder [14], schizophrenia [15], and the others [16], [17]. However, the ANT has not been used to detect possible attentional deficits among insomnia suffers.

Many studies have suggested that 24-hour hyperarousal is possibly a central pathophysiological issues in insomnia suffers [18]. Under homeostasis of sleep regulation mechanism, normal subjects showed a significant decrease in the score on Multiple Sleep Latency Test (MSLT) in responses to sleep loss with sleep deprivation, In contrast, insomnia suffers generally had normal and even increased score on MSLT after sleep loss, thus proven the hypothesis of 24-h hyperarousal in insomnia suffers [2]. Our previous study also suggest that insomnia suffers with greater score on MSLT showed more severe insomnia symptoms in the night sleep than insomnia suffers with lower score on MSLT [19]. The hyperarousal represent a condition of high activation affects somatic, cortical and cognitive functioning. Less clear is whether physiological hyperarousal affect neurophysiology performance in insomnia. Evidence from narcoleptic patients indicates that cognitive performance is influenced by daytime sleepiness [5], [20]. Other studies found that insufficient sleep and daytime sleepiness positively associated with cognitive function impairment in children [21], [22]. Therefore, daytime sleepiness reflected by a fewer score on MSLT may negatively impact on cognitive performance in primary insomnia. And, in the other hand, hyperarousal reflected by increased score on MSLT may also lead to similar effect in the evaluation of cognitive manifestation. In order to elucidate how daytime sleepiness evaluated by MSLT impact on examination of cognitive function in insomnia, we compared the ANT measures between insomnia suffers with daytime sleepiness and without daytime sleepiness in terms of score on MSLT in the present work.

## Methods

### Subjects

The study was approved by the Regional Ethical Committee of West China Hospital of Sichuan University. All patients and controls gave their informed written consent to the Regional Ethical Committee of West China Hospital of Sichuan University.

This study used a between-groups cross-sectional research design. Thirty-six primary insomnia patients (PIPs) and 26 good sleeper controls (GSCs) matched on age and sex were included in the study. PIPs were outpatients coming to the sleep medicine center. The eligibility criteria for PIPs were as follows: (1) fulfilling DSM-IV criteria [23] for primary insomnia; (2) having Pittsburgh Sleep Quality Index (PSQI) [24] score greater than 7; (3) agreeing to abstain from sedatives for at least two weeks before the start of the study. GSCs who reported no sleep complaints were recruited through advertisements. GSCs needed to satisfy the following requirements: (1) no sleep complaint; (2) satisfying PSQI score ≤7. Exclusion criteria for both groups were: (1) medical and psychiatric diseases or other sleep disorders; (2) substance abuse based on the exclusion criteria of substance abuse in DSM-IV; (3) shift work; (4) an apnea/hypopnea index (AHI)≥5 or periodic leg movements index (PLMI)>15.

### Procedures

Upon registration at the Sleep Medicine Center of West China Hospital of Sichuan University for the overnight study, participants were underwent face-to-face interview for collect general information (age, sex, education level and course of disease), Pittsburgh Sleep Quality Index, and a comprehensive assessment of daytime function with a series of questionnaires. These questionnaires including Epworth Sleepiness Scale (ESS) [25], Flinders Fatigue Scale (FSS) [26], Beck Depression Inventory (BDI) [27] and State-Trait Anxiety Inventory (SAI and TAI) [28]. The subjects were advised not to drink coffee, tea or caffeine-containing drinks, alcohol and smoking in the test day.

### Polysomnography

Overnight polysomnography (PSG) recording techniques and standard parameters were performed according to the American Academy of Sleep Medicine (AASM) Manual for the Scoring of Sleep and Associated Events [29]. Sleep data were collected via Alice 5 Diagnostic Sleep System (Philips Respironics, Bend, OR, USA). PSG recording included electroencephalogram (EEG) (F4–M1, C4–M1, O2–M1, F3–M2, C3–M2, and O1–M2), bilateral electro-oculography (EOG), electrocardiography (ECG), electromyography (EMG) (submental, anterior tibialis), oral airflow, rib cage and abdominal movements, snore microphone, and an arterial oxygen saturation sensor on the left index finger.

The observed sleep parameters included sleep onset latency (SOL), time in bed (TIB), total sleep time (TST), sleep efficiency (SE), wake time after sleep onset (WASO), microarousal index (MA index), the percentage of time (relative to TST) spent in sleep Stages 1–3 (N1–3) and in rapid eye movement sleep (REM), the AHI and the PLMS.

### Multiple Sleep Latency Test

All participants underwent MSLT following the night of PSG monitoring according to the AASM Practice Parameters for Clinical Use of the MSLT [30]. Beginning at 9:00 a.m., the test consisted of four 20-min naps spaced in 2-h interval. Sleep latency was defined as the beginning of the first 30-second epoch of stage 1 or any other sleep stage, and when no sleep occurred a score of 20 was assigned. The MSLT latency was the average of the scores from the 4 naps. The faster the participant fell asleep, the greater the inferred sleepiness.

### Attention Network Test

**Figure 1:**
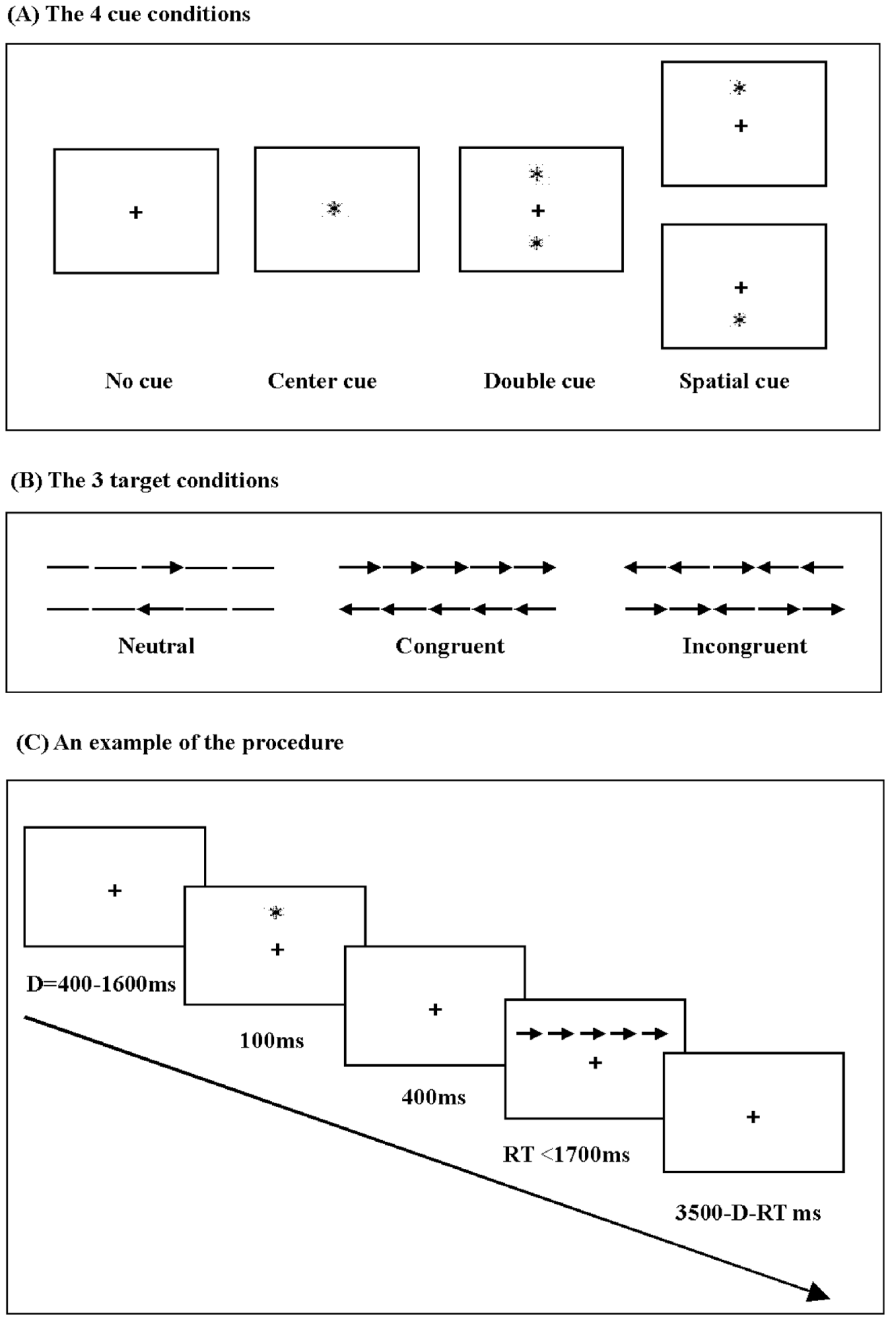
The flow chart of experimental process: (A, the four cue conditions; B, three target conditions; C, an example of the procedure). RT = reaction time.

We administered a version of the ANT [12] (see Figure 1) by E-prime software. The test consisted of a 24-trial full-feedback practice block and three experimental blocks of trials with no feedback. Each experimental block consisted of 96 trials (4 cue conditions×2 target locations×2 target directions×3 flanker conditions×2 repetitions). In each trial, after a random duration of 400–1600 ms a cue was presented for 100 ms. Four hundred ms after offset of the cue the target flankers emerged. The target flankers persisted until the participant responded or for 1700 ms if no response was given. Next, a post-target fixation displayed for duration of 3500 ms minus reaction time (RT) minus duration of the first fixation. For each trial, the participants were instructed to focus on both speed and accuracy, and to press the left or right button according to a target arrow direction. In addition, due to possible effect of hand posture on behavioral performance, we required that subjects were right-handed.

The following processing steps were performed to remove outliers: all errors RT, RT>1500 ms and RT<200 ms were removed. The three networks were calculated by subtracting RT of different conditions: alerting = no cue – double cue; orienting = central cue – spatial cue; executive control = incongruent condition – congruent condition.

### Statistical analysis

Continuous variables with normally distributed data were analyzed with Student's t-test or One-way analysis of variance (ANOVA) followed by Tukey's HSD test as the post hoc test. Otherwise, a non-parametric Mann–Whitney U-test was applied. Results are shown as the mean ± standard deviation (SD). Associations between variables were explored via Pearson Correlation Analysis. Further, Multiple Regression Analysis was performed to investigate if the sleepiness could better account for by the insomniacs' task performance adjusted for multiple confounding variables. The significance level was established at *p*<0.05. All analyses were performed using SPSS 17.0.

## Results

### Descriptive data, daytime symptoms and PSG sleep measures in PIPs and GSCs

A total of 36 PIPs and 26 GSCs were recruited in the study. There were no significant differences for demographic data concerning age, sex distribution, education years and body mass index (BMI) between the GSCs and PIPs.

Among PIPs, the illness duration of sleep difficulties was 6.48±6.0 years, 14 subjects (38.89%) reported merely sleep onset difficulties, 2 subjects (5.56%) reported only difficult in maintaining sleep, and 20 subjects (55.56%) had difficulty both in initiating and maintaining sleep. Eleven PIPs (30.56%) reported using sleep aids twice or more than twice per week, and all of them agreed to not take sedative for two weeks prior to the experiment. Result showed that the PIPs differed significantly in regard to the total score of PSQI to GSCs.

Compared to the GSCs, the PIPs had significant increase in the total scores of FSS, BDI, SAI, TAI, but not in ESS. Comparison of questionnaires revealed that PIPs reported more frequent and more severe daytime symptoms.

Controls have good sleep quality confirmed by PSG data (sleep latency less than 30 min, wake time after sleep onset <30 min, total sleep time >7 hour). The independent t-test evaluating objective sleep measures show that compared to the GSCs, the PIPs had significantly prolonged sleep latency (SOL) (20.69±19.49 vs. 7.39±5.48 min), less total sleep time (TST) (389.53±75.47 vs. 429.83±50.96 min) and fewer sleep efficiency (SE) (81.88±15.69 vs. 89.07±7.95%). In sleep architecture, the PIPs had reductions in the percentage of N3 (%TST) (17.06±9.35 vs. 21.87±8.53%) relative to the GSCs.

### ANT data in PIPs and GSCs

All participants demonstrated response accuracy over 90%. The independent t-test evaluating ANT data showed that no difference in overall RT between GSCs and PIPs. With respect to attention networks, the data revealed that PIPs had impaired executive control function compared to GSCs (87.69±39.98 vs. 69.19±27.66, *p* = 0.047), and the differences between PIPs and GSCs for alertness and orientation were not significant.

### MSLT and cognitive performance

Due to MSLT<10 min was considered as excessive daytime sleepiness (EDS) [31], the insomnia group were subdivided into PIPs with EDS (n = 12, score on MSLT<10 min) and PIPs without EDS (n = 24, score on MSLT≥10 min). As shown in Table 1, there were no significant differences for sex distribution, age, level of education, BMI and daytime symptoms between PIPs with EDS and PIPs without EDS. The group of PIPs with EDS had significantly shorter PSG-determined sleep latency than PIPs without EDS (Table 2). In ANT, PIPs with EDS had longer RT on a few different cues, targets and the overall RT, compared to PIPs without EDS. No differences were obtained among groups in the three networks (Table 3).

**Table 1:** Descriptive data and daytime symptoms in GSCs, PIPs with EDS and PIPs without EDS.

	GCSs (n = 26)	PIPs without EDS (n = 24)	PIPs with EDS (n = 12)	Group *p*
Age(years)	40.54±11.96	40.13±11.67	45.83±9.42	0.330^b^
Female (%)	61.54	58.33	58.33	0.969^a^
Education(years)	12.92±2.31	12.04±2.94	10.75±2.70	0.063^a^
BMI(kg/m^2^)	22.64±3.55	21.39±2.58	22.87±2.63	0.252^b^
PSQI	2.81±1.92	13.21±3.65	14.25±2.73	<0.001^b^^,^ c ^,^ d
FSS	5.69±4.10	12.54±5.52	13.83±6.26	<0.001^b^^,^ c ^,^ d
BDI	4.31±5.43	10.38±6.34	12.00±10.48	<0.001^a^^,^ c ^,^ d
SAI	28.27±7.10	34.33±8.43	33.33±13.66	0.062^a^
TAI	30.42±8.75	39.04±10.38	39.08±12.57	0.014^b^^,^ c ^,^ d

Female value is in %; other values are in mean ± SD;

Note: BMI = Body Mass Index; PSQI = Pittsburgh Sleep Quality Index; FSS = Flinders Fatigue Scale; BDI = Beck Depression Inventory-I; SAI = State Anxiety Inventory; TAI = Trait Anxiety Inventory.

a Kruskal-Wallis Test.

b Tukey Test;

c PIPs without EDS vs. GCSs.

d PIPs with EDS vs. GCSs.

**Table 2:** PSG sleep data in GSCs, PIPs with EDS and PIPs without EDS (mean ± SD).

	GCSs (n = 26)	PIPs without EDS (n = 24)	PIPs with EDS (n = 12)	Group *p*
SOL (min)	7.39±5.48	15.42±3.31	7.32±1.66	0.000^a^^,^ c ^,^ d
TIB (min)	483.58±48.22	481.83±43.32	470.17±31.23	0.664^b^
TST (min)	429.83±50.96	379.58±84.87	409.42±31.23	0.033^b^^,^ c
WASO (min)	46.37±41.30	76.31±86.49	50.54±27.22	0.256^a^
SE (%)	89.07±7.95	79.37±18.26	86.90±6.66	0.020^a^^,^ c
REM(% TST)	18.84±5.03	20.11±6.91	19.04±5.40	0.730^b^
N1 (% TST)	19.58±7.71	22.46±10.74	25.77±11.88	0.195^b^
N2 (% TST)	39.70±10.21	40.19±10.29	38.53±14.46	0.915^b^
N3 (% TST)	21.87±8.53	17.26±9.76	16.67±8.86	0.128^b^
MA index(n/h)	8.66±3.58	9.45±5.72	11.82±4.88	0.193^b^

Note: SOL = sleep onset latency; TIB = time in bed; TST = total sleep time; WASO = wake time after sleep onset; SE = sleep efficiency; REM = rapid eye movement Latency; MA index = microarousal index;

a Kruskal-Wallis Test.

b Tukey Test.

c PIPs without EDS vs. GCSs.

d PIPs without EDS vs. PIPs with EDS.

**Table 3:** ANT variables in GSCs, PIPs with EDS and PIPs without EDS (mean ± SD).

	GCSs (n = 26)	PIPs without EDS (n = 24)	PIPs with EDS (n = 12)	Group *p*
Overall accuracy	98.13±1.84	98.16±1.78	98.53±1.10	0.774^b^
Overall RT	750.70±132.55	720.23±93.06	831.34±161.17	0.049^b^^,^ c
Neutral				
No cue	763.02±127.55	728.19±93.88	834.74±167.83	0.062^b^
Center cue	750.14±122.98	709.06±92.63	825.29±174.30	0.036^b^^,^ c
Double cue	763.02±127.55	728.19±93.88	834.74±167.83	0.042^a^^,^ c
Spatial cue	648.32±127.06	615.83±101.45	733.14±165.70	0.038^b^^,^ c
Congruent				
No cue	769.73±138.77	733.12±103.09	847.19±173.00	0.062^b^
Center cue	757.58±152.21	725.80±97.18	833.60±170.02	0.093^b^
Double cue	744.87±143.88	703.37±98.55	818.05±190.03	0.091^a^
Spatial cue	664.66±140.56	633.10±105.13	745.27±176.82	0.073^b^
Incongruent				
No cue	850.83±151.92	838.30±117.39	938.10±160.68	0.125^b^
Center cue	830.11±156.47	804.02±95.87	918.41±139.54	0.055^b^
Double cue	808.86±148.77	804.02±95.87	902.77±165.21	0.089^b^
Spatial cue	729.29±149.48	735.80±135.70	833.84±172.17	0.114^b^
Networks				
Alerting	31.17±26.65	42.55±24.81	38.65±40.00	0.379^b^
Orienting	94.29±44.79	84.35±42.38	86.98±66.82	0.763^b^
Executive control	69.19±27.66	88.52±40.94	86.03±39.71	0.138^b^

Note: RT = reaction time.

a Kruskal-Wallis Test.

b Tukey Test.

c PIPs without EDS vs. PIPs with EDS.

**Figure 2:**
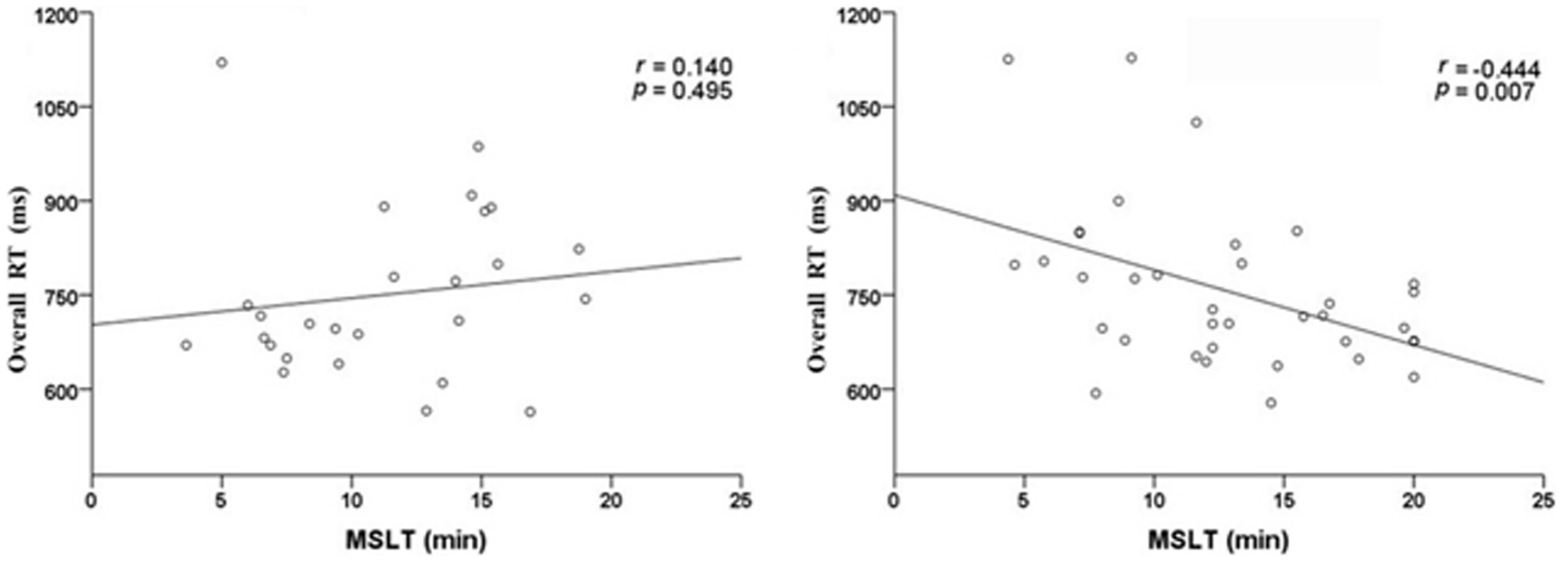
Scatter-plots demonstrating correlations for MSLT versus overall RT in good sleeper controls (left panel) and primary insomnia patients (right panel). RT = reaction time; MSLT = multiple sleep latency test.

To examine the relationship between objective sleepiness and cognitive manifestation, we performed Pearson correlation analyses for MSLT versus overall RT (Figure 2). A significant negative correlation was found among individuals with insomnia (*r* = −0.444, *p* = 0.007). But the correlation for MSLT versus overall RT was not significant in GSCs.

A multiple regression analysis was performed to determine if objective sleepiness could better account for the ANT performance adjusted for multiple confounding variables in PIPs (Table 4). Model 1 was unadjusted (*R^2^* = 0.197, *F* = 8.329, *p* = 0.007). Model 2 shows the estimated association between MSLT and overall RT controlling for only gender, age and education years (*R^2^* = 0.420, *F* = 5.620, *p* = 0.002). Model 3 additionally controls for the daytime mood factors of BDI and TAI score (*R^2^* = 0.420, *F* = 5.620, *p* = 0.002). Model 4 adds 3 PSG determined sleep parameters of sleep latency, total sleep time and sleep efficiency (*R^2^* = 0.508, *F* = 2.980, *p* = 0.014). The coefficient between MSLT latency and overall RT decreased from models 1 (coefficient = −0.444) to 4 (coefficient = −0.370), but all were significant. From models 2 to 3, the coefficient between MSLT and overall RT were all significant (coefficient = −0.349 to coefficient = −0.404).

**Table 4:** Multiple Linear Regression Models predicting overall RT in primary insomnia patients.

	Overall RT
	Beta Coefficient	95% confidence interval		*p*
Unadjusted				
MSLT latency	−0.444	−20.338	−3.530	0.007
Adjusted MSLT latency	−0.370	−19.438	−0.480	0.040
Age	0.375	0.723	7.953	0.021
Sex				
Female	0.395	17.365	186.781	0.020
Education years	0.054	−14.416	19.275	0.769
BDI	−0.396	−14.885	1.848	0.121
TAI	0.272	−2.940	9.103	0.303
Sleep latency	−0.086	−3.679	2.535	0.708
Total sleep time	−0.283	−1.904	0.935	0.489
Sleep efficiency	0.447	−3.353	10.715	0.292

Note: RT = reaction time; BDI = Beck Depression Inventory-I; SAI = State Anxiety Inventory; MSLT = multiple sleep latency test.

Model 1: unadjusted;

Model 2: adjusted for gender, age and education years;

Model 3: adjusted for gender, age, education years, BDI and TAI;

Model 4: adjusted for gender, age, education years, BDI, TAI, sleep latency, total sleep time and sleep efficiency.

## Discussion

### Attention networks

This study aims to investigate the attention function of primary insomnia and its associated factors. Fan et al. [12] verified the three attention networks were independent, although the overall RT did have correlation with the conflict scores measuring executive control. They proposed that the executive control network is the most reliable component of the three attention networks. Our results showed that patients with insomnia had impairment in executive function, but they displayed normal range in alerting, orienting and overall RT.

Executive functioning has been formulated in a variety of ways over the past few years. It represents advanced cognitive processes such as planning, decision-making, and mainly relates to resolving conflict [32], [33]. Our data showed that patients with insomnia express the impairment in executive function, which would support the notion that the performance decline is associated with increasing cognitive demands of insomnia [34].

Lines of evidences from functional neuroimaging studies indicate that executive function mainly involves the prefrontal and the anterior cingulate cortices [35], [36]. Functional metabolic researches demonstrate that the regions associated with executive function are sensitive to sleep deprivation [37]. Executive function is probably largely influenced by dopamine system [38], [39], since dopamine has been proposed as a mechanism for promoting arousal [40]. Altena et al. reported hypoactivation of the prefrontal cortex by functional Magnetic Resonance Imaging (fMRI) in insomnia sufferers relative to controls, but in the absence of a behavioral deficit [10]. Given the findings from the neuroimaging results, broader neuropsychological tests would be necessary to assess executive network in insomnia, yet the area has received much less attention. Several early findings showed that patients with insomnia did not differ from healthy controls on various executive tasks [10], [41]–[43]. One study found that insomniac scored lower than controls on a task of logical reasoning [44]. Fernandez-Mendoza et al. [45] found only that insomniacs with short sleep duration were impaired on tasks tapping of executive control for attention. Overall, despite consistent neuroimaing findings that insomnia may influence prefrontal cortex which may be related to executive control, the objective evidence for impairment in executive function in insomnia is contradictory.

### Objective daytime sleepiness and cognitive performance

The current investigation was conducted to explore the relationship between objective sleepiness and attention function in insomnia sufferers. In the present study, insomniacs with objective daytime sleepiness did not show more severe daytime symptoms, but performed worse on objective neuropsychological performance. Correlations analysis revealed a significant negative association between MSLT and overall RT in insomnia patients, but not in controls. Multiple linear regressions consistently confirmed that insomnia sufferers who had lower score of MSLT tended to have longer overall RT. However there were no associations between the three attention networks (alerting, orienting, and executive control) and MSLT latency. The significant longer overall RT of insomnia with daytime sleepiness may reflect a global impairment of attention, but not in certain attention networks. In addition, we did not find any significant associations between PSG evaluated sleep parameters and objective cognitive performance.

The studies with sleep deprivation have consistently reported that increasing daytime sleepiness lead to cognitive impairment [37], [46], [47]. Unlike to sleep deprivation, results from insomniacs did not find consistent decline of cognitive performance. Thus, the 24-h hyperarousal in insomnia may play as a compensatory function to cognitive manifestation. Consistent to the viewpoint, our finding provide evidence that individuals with insomnia who have higher arousal levels in terms of MSLT scores have less cognitive impairment. Limited research has focused on the relationship between sleepiness and cognitive performance in insomnia patients. Covassin et al. [48] found that subject sleepiness (Stanford Sleepiness Scale, SSS) was negatively correlated with test accuracy in insomnias. Another group found that MSLT latency was negative associated with once cognitive performance [49]. However, Lamond et al. [50] reported that cognitive impairments persisted when subjective sleepiness recovered. As mentioned above, objectively measured daytime sleepiness may play an important role in assessment of cognitive function of insomnia patients. Further studies are needed to explore whether medications that promote wakefulness can improve the cognitive functions in insomnia.

### Limitations

Several limitations of the current study should be addressed. The primary limitation was small sample size. Second, the PIPs group comes from a heterogeneous population including patients on medication; different subtypes of insomnia (sleep initiating problems and sleep maintenance problems). Additionally, the present study did not address factors such as IQ and socioeconomic status that may affect cognitive performance.

## Conclusions

Our results confirm that primary insomnia sufferers show attention deficits of the executive network. The major finding of this study supports that there is significant negative correlation between cognitive manifestation and sleepiness in terms of the MSLT measure among primary insomnia suffers. These results add novel data to the literature by suggesting that 24-hour hyperarousal potentially plays a key role in the cognitive performance of insomnia patients. More importantly, it indicates that clinical reductions of objective sleepiness may improve cognitive function in insomnia suffers.
